# Outcomes of Surgical Repair for Persistent Truncus Arteriosus from Neonates to Adults: A Single Center's Experience

**DOI:** 10.1371/journal.pone.0146800

**Published:** 2016-01-11

**Authors:** Qiuming Chen, Huawei Gao, Zhongdong Hua, Keming Yang, Jun Yan, Hao Zhang, Kai Ma, Sen Zhang, Lei Qi, Shoujun Li

**Affiliations:** Pediatric Cardiac Surgery Center, National Center for Cardiovascular Disease and Fuwai Hospital, Chinese Academy of Medical Sciences, Peking Union Medical College, Beijing, PR China; Scuola Superiore Sant'Anna, ITALY

## Abstract

**Objective:**

This study aimed to report our experiences with surgical repair in patients of all ages with persistent truncus arteriosus.

**Methods:**

From July 2004 to July 2014, 50 consecutive patients with persistent truncus arteriosus who underwent anatomical repair were included in the retrospective review. Median follow-up time was 3.4 years (range, 3 months to 10 years).

**Results:**

Fifty patients underwent anatomical repair at a median age of 19.6 months (range, 20 days to 19.1 years). Thirty patients (60%) were older than one year. The preoperative pulmonary vascular resistance and mean pulmonary artery pressure were 4.1±2.1 (range, 0.1 to 8.9) units.m^2^ and 64.3±17.9 (range, 38 to 101) mmHg, respectively. Significant truncal valve regurgitation was presented in 14 (28%) patients. Hospital death occurred in 3 patients, two due to pulmonary hypertensive crisis and the other due to pneumonia. Three late deaths occurred at 3, 4 and 11 months after surgery. The actuarial survival rates were 87.7% and 87.7% at 1 year and 5 years, respectively. Multivariate analysis identified significant preoperative truncal valve regurgitation was a risk factor for overall mortality (odds ratio, 7.584; 95%CI: 1.335–43.092; p = 0.022). Two patients required reoperation of truncal valve replacement. One patient underwent reintervention for conduit replacement. Freedom from reoperation at 5 years was 92.9%. At latest examination, there was one patient with moderate-to-severe truncal valve regurgitation and four with moderate. Three patients had residual pulmonary artery hypertension. All survivors were in New York Heart Association class I-II.

**Conclusions:**

Complete repair of persistent truncus arteriosus can be achieved with a relatively low mortality and acceptable early- and mid-term results, even in cases with late presentation. Significant preoperative truncal valve regurgitation remains a risk factor for overall mortality. The long-term outcomes warrant further follow-up.

## Introduction

Persistent truncus arteriosus (PTA) is a congenital malformation that is relatively uncommon for about 2–4% of all congenital heart defects [1]. Anatomically, a single great artery arising from the base of the heart supplies the aorta, pulmonary arteries and coronary arteries. Initially, PTA repair had high mortality [2]. Palliation with pulmonary artery banding followed by total correction at a later age is currently not believed to have any advantages and is associated with high mortality [3]. Marked improvements of pediatric cardiac surgery and cardiology in recent decades has led to earliest possible complete repair [4–6]. In children presenting later to the hospital, surgery may be complicated, or even contraindicated, by presence of elevated pulmonary vascular resistance. Therefore, we report a single-center experience with complete repair of PTA in patients of all ages, from neonates to adults.

## Methods

A retrospective review was performed of 50 consecutive patients who underwent anatomical PTA repair from July 2004 to July 2014 at Fuwai Hospital, Beijing, China. Approval for the study was obtained from Institutional Review Board of Fuwai Hospital with patient consent waived. And patient information was anonymized and de-identified prior to analysis. Medical records were reviewed for demographic data, preoperative functional status, operative procedure, preoperative and postoperative hemodynamics, and early and midterm survival.

All the patients underwent preoperative chest radiography, electrocardiogram, arterial blood gas estimation and echocardiography. Cardiac catheterization was performed under general anesthesia in order to obtain hemodynamic data in patients older than 3 months. Preoperative pulmonary artery pressure and pulmonary vascular resistance index (PVRi) were measured by using the conventional cardiac catheterization protocol and Fick method. The operability of PTA with increased PVRi who presented late was determined with history, physical examination, chest radiography, electrocardiogram, echocardiography and hemodynamic data obtained from cardiac catheterization. Criteria for inoperability were the same as for patients with ventricular septal defect (VSD). When PVRi is severely elevated (greater than 8 units.m^2^), surgery is inadvisable.

### Surgical management

Except for the second patient of this series who had palliated pulmonary banding over ten months, all other 49 patients underwent one-stage anatomical repair. Operative repair was performed though a standard median sternotomy. Cardiopulmonary bypass was established with bicaval and ascending aortic cannulation at moderate hypothermia. The patent ductus arteriosus was ligated or cut off if existence. Surgical repair of PTA consisted of VSD closure with a Dacron patch, harvesting of pulmonary arteries, reconstruction of the aorta, and reconstruction of the right ventricle outflow tract (RVOT). The criteria for leaving the atrial septum open was the pulmonary artery pressure > 80% of systemic pressure measured in the operating room before cardiopulmonary bypass. The pulmonary arteries were excised from the ascending aorta through careful dissection to avoid injury to the truncal valve or coronary artery. The resultant aorta defect was directly closed or with autologous pericardial patch or bovine pericardial patch. The truncal valve was inspected to determine the morphology and function and the appropriate repair procedure was performed if required. Surgical techniques were individualized to the patient’s valve pathology on the basis of the operating surgeon’s opinion. Truncal valve repair consisted of a combination of different methods: commissuroplasties, partial suture of a wide commissure and complete suture of one commissure. Indications for truncal valve replacement were severe truncal valve regurgitation when valvuloplasty failed.

Right ventricle-pulmonary artery continuity was established with an aortic or pulmonary homograft, a bovine jugular venous valve conduit. Conduit size was determined according to the calculated Z value for each implanted valve by using the valve diameter compared with the normal value. The conduit was anastomosed to the infundibulotomy either directly or by constructing a hood to “roof-over” the anterior portion of the anastomosis to prevent conduit distortion. In patients without left pulmonary artery, the bovine jugular venous valve conduit was connected only to the right pulmonary artery.

Additional procedures performed at the time of repair included repair of interrupted aortic arch, partial anomalous pulmonary venous connection, and mitral valvuloplasty when mitral regurgitation was ≥moderate. Intraoperative transesophageal echocardiography was used in all patients and revealed satisfactory repair in all without any unintended residual septal defects.

### Postoperative care

When the patients were transferred back in the intensive care unit, routine deep sedation with continuous sedative intravenous infusion and neuromuscular blockade was practiced. All patients were ventilated with the partial oxygen pressure maintained at 100 to 120 mmHg and the partial arterial carbon dioxide pressure maintained 35 to 45 mmHg. Intravenous dopamine (3 to 10 μg/kg/min) and milrinone (0.25 to 0.75 μg/kg/min) were used for hemodynamic management. The pulmonary artery pressure was monitored occasionally though echocardiography in the postoperative course. Inhaled nitric oxide was routinely used. Pulmonary hypertensive crisis were prevented with drugs such as sildenafil and Bosentan, as appropriate. The target dose of Bosentan was 31.5–125 mg *b*.*i*.*d*. according to weight. Patients <10 kg in weight were given 15 mg *b*.*i*.*d*. Sildenafil was added as required when patients deteriorated on Bosentan monotherapy.

### Data collection and definitions

Hospital death was defined as the mortality within 30 days of operation or during the same hospital admission. Mortality after that time was considered as late death. Pulmonary arterial hypertension (PAH) was defined as mean pulmonary artery pressure more than 25 mmHg. For analysis, truncal valve regurgitation was considered “significant” when graded as moderate or severe.

### Statistical analysis

Statistical analyses were performed with the SPSS version 19.0 for Windows (SPSS, Chicago, IL). Data are presented as mean ± SD or median (range) where appropriate. Continuous variables were analyzed with Student’s t-test for normally distributed variables, or the related samples Wilcoxon signed-rank test for non-normally distributed variables, and categorical variables were analyzed by using Chi-square test or Fisher’s exact test. Actuarial survival was calculated by using the Kaplan-Meier method. The time-related outcomes were estimated using Kaplan-Meier analysis with a log-rank test to evaluate difference. Risk factors with p values of less than 0.2 after univariate analysis or those judged to be clinically important were considered eligible for entering into the multivariate analysis. Potential risk factors for overall deaths were analyzed with Cox’s proportionate hazard models using a forward conditional stepwise method. All statistical tests were two-tailed and p<0.05 was taken as significant.

## Results

### Preoperative characteristics

Fifty consecutive patients (32 male and 18 female) were identified. The preoperative characteristics are summarized in Table 1. The median age at the time of repair was 19.6 months, with a range of 20 days to 19.1 years, including 2 neonates (<1 month), 18 infants(1 month—1 year), 28 children (1–18 years) and 2 adult (>18 years). Most of them were late referral for various reasons. At the moment of admission, all of them had controlled cardiac failure and none of them needed preoperative ventilation. The preoperative PVRi and mean pulmonary artery pressure were 4.1±2.1 (range, 0.1 to 8.9) units.m^2^ and 64.3±17.9 (range,38 to 101) mmHg. Only one patient’s PVRi was higher than 8 units.m^2^. According to the classification of Collett and Edwards, 33 patients were type I, 16 were type II, the remaining one patient was type III with non-confluent pulmonary arteries. Truncal valve regurgitation was absent or trivial in 14 patients, mild in 22, moderate in 13, and severe in the remaining one patient. There were 3 patients with mild truncal valve stenosis, 2 patients with moderate stenosis according to the peak instantaneous truncal valve systolic gradients by Doppler examination. The number of truncal valve leaflets was variable: 41 had three leaflets, 4 had four leaflets, and 5 had two leaflets. Associated anomalies were listed in Table 2.

**Table 1:** Patient characteristics.

	Overall (n = 50)	Age<1 year(n = 20)	Age≥1 year(n = 30)	P
Median age at surgery(m)	19.6(20d to 229.5)	7.4(0.7–10.8)	54.5(14.7–229.5)	<0.001
Median weight (kg)	9.6 (2.7 to 46)	5.6(2.7–8.0)	13.8(7.5–46)	<0.001
Male (%)	32 (64%)	10(50%)	22(73.3%)	0.134
Anatomic classification				0.069
I	33 (66%)	10(50%)	23(76.7%)	
II	16 (32%)	10(50%)	6(20%)	
III	1 (2%)	0	1(3.3%)	
SaO_2_ (%)	89.2±5.8	87.5±6.8	89.8±4.8	0.200
LVEF	67.5±7.2	69±7.9	66.5±6.7	0.227
Mean PAH^†^	64.3±17.9	49.5±8.1	71.4±17.1	**<0.001**
Pulmonary valve resistance^†^	4.1±2.1	3.6±2.6	4.3±1.7	0.342
Truncal valve regurgitation				0.353
absent or trivial	14(28%)	7(35%)	7(23.3%)	
mild	22(44%)	9(45%)	13(43.3%)	
moderate	13(26%)	4(20%)	9(30.0%)	
severe	1(2%)	0	1(3.3%)	
RV-PA Conduit Z score	2.3 ± 1.3	2.8 ±0.8	1.99 ±1.4	**0.030**
CPB time (min)	149.3±45.4	135.5±39.8	157.8±46.4	0.090
Cross clamp time (min)	107.2±29.3	91.9±19.6	116.9±30.5	**0.003**
Mechanical ventilation time(hour)	141.2±248.2	178.2±312.9	117.7±199.3	0.412
Intensive care unit stay (days)	10.6±13.0	16.7±23.0	8.7±10.7	0.161
Total length of stay (days)	18.9±16.9	22.9±22.9	16.3±10.9	0.243
Hospital death	3 (6%)	2(10%)	1(3.3%)	0.556
Late death	3 (6%)	2(10%)	1(3.3%)	0.556
Reoperation	3 (6%)	1(5%)	2(6.7%)	1.000

^†^: included 44 patients beyond 3 months old.

CPB: cardio-pulmonary bypass; d: days; m: months; PA: pulmonary artery; PAH: Pulmonary artery pressure; RV: right ventricle; RVOT: right ventricular outflow tract; SaO_2_: saturation O_2_; y: years

**Table 2:** Associated anomalies.

Anomaly	Patients (N = 50)	
	N	%
Secundum atrial septal defect/PFO	13	26
Right aortic arch	15	30
Patent ductus arteriosus	2	4
Persistent left superior vena cava	4	8
Hypoplasia of the left lung	1	2
Congenital absence of left pulmonary artery	1	2
Interrupted aortic arch	1	2
Coronary artery anomaly	4	8
Abnormal ostial position	2	4
Single coronary artery	2	4
Coarctation of the aorta	1	2
Partial anomalous pulmonary venous connection	1	2
Truncal valve stenosis ≥Moderate to severe	2	4
Mitral regurgitation ≥moderate	3	6

PFO: Patent Foramen Ovale

### Early results

The mean cross-clamp and cardio-pulmonary bypass times were 107.2±29.3 and 149.3±45.4 minutes, respectively. Seven of the fourteen patients with significant truncal valve regurgitation underwent truncal valve repair and one adult received truncal valve replacement after unsuccessful valvuloplasty. Right ventricle-pulmonary artery continuity was established with an aortic (n = 11) or pulmonary homograft (n = 8) in 19 patients (range, 16–26 mm), and a bovine jugular venous valve conduit in 31 patients (range, 10–22 mm). The atrial septal defect or patent foramen ovale was left open in five patients.

There were three hospital deaths (6.0%, 3/50) which occurred at 8, 4 and 77 days after surgery, respectively. The detailed deaths were listed in Table 3. Hospital deaths have been attributed to the following causes: uncontrollable pulmonary hypertensive crises in two patients and bacterial pneumonia in one. Two of them had complex lesions, such as hypoplasia left lung and coronary anomalies. The following variables were analyzed to assess if they were risk factors for hospital mortality: age, age of more than 1 year, gender, weight, preoperative PVRi and mean pulmonary artery pressure, significant truncal valve regurgitation, presence of coronary artery anomalies, RVOT techniques, cardio-pulmonary bypass time and cross-clamp time. None of them was detected as the risk factor of early death on univariate analysis.

**Table 3:** Hospital death and late deaths after repair of persistent truncus arteriosus.

	sex	Age (mo)	PVRi (wood units.m^2^)	mPAP (mmHg)	Truncal valve regurgitation	Concomitant anomaly	Interval from operation to death	Cause of death
1^a^	F	62.4	6.1	66	Moderate	NA	7 days	Pulmonary hypertensive crisis
2^a^	M	8	8.9	64	Mild	Hypoplasia left lung	4 days	Pulmonary hypertensive crisis
3^a^	M	2.2	NA	NA	Moderate	Bicuspid TV, abnormal coronary ostial position	77 days	bacterial pneumonia
4^b^	F	7.8	2.3	42	Moderate	Quadricuspid, moderate stenosis TV	4 mo	Sudden death
5^b^	M	10.2	4.8	38	Mild	NA	3 mo	bacterial pneumonia
6^b^	M	49.9	3.4	66	Moderate	NA	11 mo	Sudden death

^a^: Patient were hospital deaths

^b^: Patient were late deaths

F: female; M: Male; mo: months; mPAP: mean pulmonary artery pressure; NA: not applicable; PVRi: pulmonary vascular resistance index; TV: truncal valve;

Sternal closure was delayed for 3 days in one patient for hemodynamic liability. Two patients needed reoperation for hemostasis. Minor adverse events included pleural effusions requiring drainage in 2 patients, pericardial effusion requiring drainage in 1, and prolonged ventilation of more than 7 days in 9 (5 required reintubation). Thirty five patients (70.0%) had an uneventful postoperative course. The oxygen saturations ranged from 95% to 100% at the time of discharge. Moderate-to-severe regurgitation of truncal valve was not noted in all survivors before discharge. Seven patients had moderate pulmonary regurgitation. The mean gradient between the right ventricle and the pulmonary artery was 18.7±10.9 (range, 5–47) mmHg.

### Late results

No patient was lost to follow-up. Patients were followed up to a median of 3.4 years (range, 3 months to 10 years). Three late deaths occurred at 3, 4 and 11 months after surgery, one due to bacterial pneumonia, and the other two due to sudden death. The probability of patient survival (Kaplan-Meier) was 87.7% and 87.7% at 1 and 5 years, respectively.

**Fig 1:**
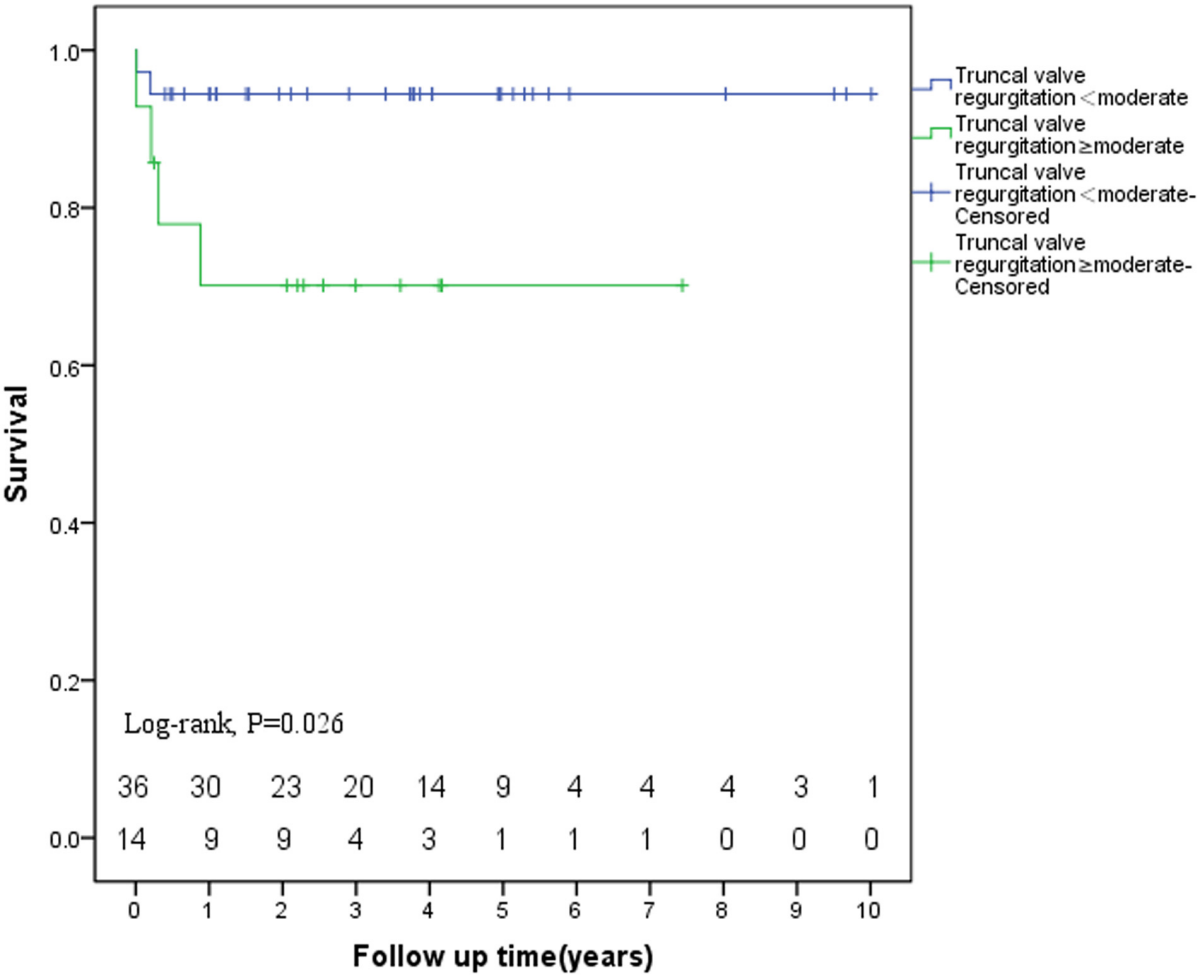
The Kaplan-Meier estimate of survival stratified by truncal valve regurgitation.

The Kaplan-Meier survival analysis stratified according to preoperative truncal valve regurgitation showed that significant preoperative truncal valve regurgitation (greater than or equal to moderate truncal valve regurgitation) was a risk factor for overall death (Log rank, p = 0.026, Fig 1). Multivariate analysis identified significant preoperative truncal valve regurgitation was a risk factor for overall mortality (odds ratio, 7.584; 95%CI: 1.335–43.092; p = 0.022) (Table 4).

**Table 4:** Risk factors for overall death.

Risk factors	P value (univariate)	P value (multivariate)	HR (95%CI)
Age at surgery	0.282		
Age > 1 year	0.134	0.070	5.044(0.876–29.043)
Weight (kg)	0.307		
Truncal valve regurgitation ≥moderate	**0.026**	**0.022**	**7.584(1.335–43.092)**
Coronary abnormalities	0.267		
Mean pulmonary artery pressure	0.229		
Pulmonary vascular resistance	0.355		
RVOT techniques	1.000		
Aortic cross-clamp time	0.392		
Cardio-pulmonary bypass time	0.517		

HR: Hazard ratio; CI, confidence interval; RVOT: right ventricular outflow tract

During the follow-up period, among 47 hospital survivors, only 3 patients (6.4%) required reoperation. One patient underwent Bentall procedure for ascending aortic dilation (60mm) and perivalvular leakage 5.9 years later after initial truncal valve replacement. Another patient underwent truncal valve replacement for severe truncal valve regurgitation two years after PTA repair. One patient underwent conduit replacement for bovine jugular venous valve conduit stenosis two years after repair. The Kaplan-Meier estimate of freedom from reoperation was 92.9% at 5 years.

At latest examination, there was one patient with moderate-to-severe aortic valve regurgitation, four with moderate and the remaining were trivial or mild. Thirteen patients had moderate pulmonary regurgitation. The mean peak gradient between the right ventricle and the pulmonary artery was 21.4±19.8 mmHg (range: 9 to 74 mmHg). A residual left-to-right shunt was present in 5 children, and all were trivial by echocardiography. Three patients had residual PAH at the latest echocardiography (34, 39, and 51mmHg). One of them was taking Bosentan for severe PAH. The mean left ventricle ejection fraction was 64.3±8.4% (range, 52–73). All survivors were in NYHA class I-II.

### Late referral

Sixty percent of the patients were operated after the age of one year due to late referral. The preoperative, operative and follow-up data were compared between the two groups (Table 1). The preoperative mean pulmonary artery pressure and cross clamp time of patients older than one year was significantly higher than that of patients younger than one year (P<0.001). Relatively larger conduits were inserted in younger patients (P = 0.03). There was no statistical difference in the postoperative variables, but younger patients tended to have longer intensive care unit stay (P = 0.161). Mortality did not differ between the two groups. No significant difference was presented in follow-up results.

During the same period, a total of 19 patients were recorded as admitted to the hospital and judged inoperable. The median age of these patients was 3.9 years old (range, 1.3 to 12.1 years). Cyanosis and dyspnoea on exertion were the most common symptom. Sixteen patients were type I and the remaining three were type II based on Collette and Edwards’ classification. Truncal valve regurgitation was absent or trivial in 16 patients and mild in 3 patients. The median left ventricle ejection fraction was 59% (range: 54–68%). The mean PVRi and mean pulmonary artery pressure were 15.3 ± 4.7 (range, 9.1 to 28.6) units.m^2^ and 85.2 ± 16.8 (range, 67 to 118) mmHg. Their pulmonary vasculature parameters showed no or weak reactivity to oxygen, and they were deemed inoperable. Majority of them were lost to follow-up. Only five patients were follow-up with telephone at the median age of 5 years. All of them were on medical treatment, including fluid restriction and Bosentan or sildenafil, in NYHA class III.

## Discussion

This retrospective study encompassed a 10-year period experience and a true evolution in the understanding of the pathophysiology of this defect. We found that late complete repair of PTA was feasible in late referral patients with no significant difference in postoperative morbidity and mortality when compared with that of timely repair in infancy. The first successful surgical repair of PTA was achieved by McGoon et al. in 1967 [7]. Rapid advancement in pediatric cardiac surgery and cardiology in recent decades have led to early corrective surgery in much of the developed world [4–6]. However, in developing countries, due to a previous lack of opportunity to close defects during infancy, PAH in children with congenital heart disease is common. This situation is now becoming a current issue, as health care in these countries is starting to improve. Delaying the operation of PTA increases the risk of pulmonary vascular obstructive disease. Without surgical treatment, at least 80% of these patients died before 1 year of age [8].

There are rare untreated patients surviving to adult age, and nearly all of them have severe pulmonary vascular obstructive disease or Eisenmenger syndrome [9–11]. Neonates had high PVRi that gradually decreased after birth. As they grew older, lower PVR resulted in excessive pulmonary blood flow, causing congestive heart failure. The reason for survival beyond infancy was mostly due to an increase in the PVRi, which relieved the left ventricle volume overload but at the cost of increase in cyanosis. In addition, pulmonary artery hypoplasia might induce protection of the pulmonary circulation and thus might be beneficial. In this cohort, some patients had survived till adolescence or even adults, probably due to a slowly progressive increase in PVRi, which was confirmed by cardiac catheterization. They were at high risk for pulmonary vascular occlusive disease. Once irreversible pulmonary vascular obstructive disease is established, complete repair of PTA may be problematic because of right ventricular failure and postoperative pulmonary hypertensive crisis. In our study, 60% of them were diagnosed after one year of age because of late referral. Compared with the patients younger than one year of age, patients older than one year of age had less major associated anomalies, such as coronary artery anomalies, anomalous pulmonary venous connection, left lung hypoplasia or absence of left pulmonary artery. Most of them were not in severe clinical condition, but symptomatic with congestive heart failure as growing up. The age older than 1 year old was not risk factor in this cohort. Probably late referral patients were selected by the nature and thus with less anomalies and with a comparable outcome to early referral.

There were challenges in late referral patients with regard to assessment of operability without consensus. The PVRi of 6–8 wood units.m^2^ was widely accepted as a cut-off for operability in children with large left to right shunts [12]. Our patients’ PVRi were similar to those seen in one study from Turkey [13] which reported surgical repair experience with 7 patients with a mean PVRi of 9.04 ± 4.2 wood units.m^2^ beyond the first year of life. However, assessing operability only on the basis of cardiac catheterization might be misleading. Although cardiac catheterization measurements of hemodynamics were helpful, and was indeed the best tools available at present, they were not completely reliable. In our experience, medical history, physical examination, chest radiograph, arterial blood gas estimation, electrocardiogram and echocardiography were important to provide independent useful information on the operability. Clinically evident cyanosis was a strong predictor of inoperability. The chest radiograph suggested inoperability with normal heart size, normal or reduced vascularity.

Before the present study, few studies had reported the management of right ventricular dysfunction from long-standing PAH and increased risk of postoperative pulmonary hypertensive crisis after surgical repair of patients with PTA who were late referrals [9–10, 14]. Some authors [15–16] reported flap valve double patch or a unidirectional flap valve VSD patch to allow right-to-left shunting and decompression of the right ventricle during periods of increased PVRi. Similarly, we maintained patency of the foramen ovale or performed atrial septum defect patch fenestration to prevent pulmonary hypertensive crises. Moreover, special treatment must be given after surgery because of the possible postoperative pulmonary hypertensive crisis. In our study, patients were routinely kept under deep sedation with continuous sedative and neuromuscular blockade. The postoperative pulmonary artery pressure was monitored occasionally though echocardiography. Inhaled nitric oxide, oral sildenafil or Bosentan were used to manage pulmonary hypertensive crisis. In the latest follow-up, there were three patients (6.8%, 3/44) with residual PAH, only one needed medication.

Another issue of particular importance in this subset of patients was the management of the truncal valve. For decades, the most important factor contributing to early death was moderate-to-severe truncal valve regurgitation in patients with PTA, irrespective of whether they underwent early repair. The optimal approach to manage truncal valve regurgitation was controversial. A mild to moderate regurgitation was well tolerated postoperatively after reduction of the volume overload. Our study agreed with a number of previous reports by showing that significant truncal valve regurgitation was a significant risk factor for overall mortality. A variety of truncal valve repair techniques had been reported [17–18] such as suture valvuloplasty, leaflet excision, commissural suspension, and remodeling techniques, which were preferred for effective and durable. However, in extreme cases, valve replacement might be inevitable. McElhinney et al [19] reported 66% early mortality in truncal valve replacement, and 25% early mortality in truncal root replacement with a homograft. In the present series, only one patient underwent initial truncal valve replacement for severe truncal valve regurgitation. Patrick et al. showed that neonates and adults had a significantly higher truncal valve reoperation rate than did children [18]. But we didn’t find age to be a risk factor for truncal valve reoperation. Moreover, Roland et al. also reported that age was not an increased risk factor for reoperation after truncal valve replacement [17].

The ideal right ventricle to pulmonary artery conduit remains controversial, especially in high-pressure systems. Homograft was the first choice for RVOT reconstruction because of good hemodynamic characteristics and excellent tissue-handling properties [20]. However, the use of small homograft was limited by the lack of donors. Baskett and his associates had identified PAH as a main risk factor for homograft failure [21]. In our series, only one patient had bovine jugular venous valved conduit replacement for stenosis two years after repair. None of the homografts required revision or replacement despite PAH. The choice of conduit was individualized for each patient, based on the conduit availability and underlying anatomy. Oversizing conduits (z valve: 2.3 ± 1.3) were used in high-pressure system to prolong the conduit life. This was also consistent with data reported by Fiore and associates [22], as well as others [13, 23]. Furthermore, Joyce and his associates [24] reported that smaller conduit size was a significant factor associated with early conduit reoperation.

This study should be interpreted in light of its limitations. It was a retrospective study, not prospective or randomized. The statistical results should be interpreted with caution for the small size of the cohort which may influence the stability of Cox’s proportional hazard models. The operations were performed by different surgeons and the decision for operability was determined according to the patient’s anatomy, surgeon preference and evolved management of PAH with complex congenital heart disease. Furthermore, late referral patients were already selected by the nature and thus with less anomalies and with a comparable outcome. However, PTA was an uncommon congenital heart disease and case series with late repair were important for expanding our knowledge of this anomaly. Nevertheless, larger series have not been reported.

## Conclusions

In conclusion, our experience suggest that complete repair of PTA can be achieved with a relatively low mortality and acceptable early- and mid-term results, even in case with late presentation. Significant preoperative truncal valve regurgitation remains a risk factor for overall mortality. The long-term outcomes warrant further follow-up.
